# Progranulin Is a Useful Biomarker to Predict Mortality in ICU Patients with Low Burden of Organ Dysfunction

**DOI:** 10.3390/biomedicines14040744

**Published:** 2026-03-24

**Authors:** Jochen Johannes Schoettler, Lutz Pridzun, Bertram Flehmig, Holger A. Lindner, Verena Schneider-Lindner, Joerg Krebs, Franz-Simon Centner, Manfred Thiel

**Affiliations:** 1 Department of Anesthesiology, Surgical Intensive Care Medicine and Pain Medicine, Medical Faculty Mannheim, University of Heidelberg, Theodor-Kutzer-Ufer 1-3, 68167 Mannheim, Germany; jochen.schoettler@umm.de (J.J.S.); holgera.lindner@medma.uni-heidelberg.de (H.A.L.); verena.schneider-lindner@medma.uni-heidelberg.de (V.S.-L.); joerg.krebs@umm.de (J.K.); franz-simon.centner@umm.de (F.-S.C.); 2 Mediagnost, Diagnostics Research and Development GmbH, Aspenhaustr. 25, 72770 Reutlingen, Germany; pridzun@mediagnost.de (L.P.)

**Keywords:** progranulin, critical illness, mortality prediction, organ dysfunction

## Abstract

**Background/Objectives**: Early prognostication in critically ill patients with low burden of organ dysfunction (BOD) remains challenging. Progranulin (PGRN), a hypoxia inducible and anti-inflammatory protein, may offer prognostic value. We investigated whether PGRN levels predict mortality in ICU patients stratified by their BOD. **Methods**: In this secondary analysis of a prospectively recruited ICU cohort (*n* = 99), patients were categorized into low (Sequential Organ Failure Assessment Score (SOFA) ≤ 8) and high (SOFA > 8) BOD groups. Plasma PGRN concentrations were measured every 8 h for up to 5 days. Initial values and kinetic parameters (maximum, mean, and normalized area score (NAS)) were evaluated. Associations with in-hospital mortality were analyzed by univariate logistic regression and area under the receiver operating characteristic curve (AUROC) comparisons. **Results**: In patients with low BOD (*n* = 53), the PGRN kinetics were significantly associated with in-hospital mortality, with odds ratios of 1.086 (95% CI 1.027–1.148), 1.102 (95% CI 1.025–1.184), and 1.093 (95% CI 1.021–1.170) for maximum, mean, and NAS values, respectively. The respective AUROC values were 0.815 (*p* = 0.001), 0.753 (*p* = 0.010), and 0.738 (*p* = 0.016). By contrast, none of the PGRN metrics predicted mortality in patients with high BOD (*n* = 46; all AUROC values < 0.61, *p* > 0.25). The respective SOFA and CRP metrics were not predictive in low BOD patients. Maximum PGRN levels predicted death at least 32 h in advance. **Conclusions**: Serial PGRN measurements offer prognostic information, particularly in ICU patients with low BOD, a group whose deterioration is often difficult to anticipate and may be underestimated by conventional scoring systems such as SOFA. These findings support further investigation of PGRN as a tool for early risk stratification in critical illness.

## 1. Introduction

Early identification of critically ill patients with an unfavorable prognosis is essential for initiating timely diagnostic and therapeutic interventions that may improve survival outcomes [1]. Microcirculatory damage caused by inflammatory processes, whether due to infectious or non-infectious diseases [2], can lead to organ dysfunction and failure by impairing perfusion-dependent oxygen delivery and causing mitochondrial dysfunction [3]. Tissue hypoxia and dysoxia hence are among the first pathogenetic events of organ dysfunction resulting in lethal multiple organ failure [3]. Detecting patients at high risk of death as early as possible is essential for initiating intensified treatment strategies.

Progranulin (PGRN) is a multifunctional protein involved in various biological processes, including embryogenesis [4,5], neurodegenerative diseases [6,7], tumorigenesis [8,9], and tissue repair [10]. It plays an immunomodulatory role in anti-inflammatory responses and contributes to host defense mechanisms against bacterial [11] and fungal [12] infections. PGRN has been shown to be upregulated in myelopoietic cells [12], and particularly in fibroblasts [13] and neuroblastoma cells [14] in response to hypoxic stress. Expression levels of hypoxia inducible factor 1-alpha (HIF-1α) are correlated positively with PGRN protein expression in vitro [15] and in vivo [16], suggesting the regulation by hypoxia-sensitive mechanisms. As a mismatch in oxygen demand and supply plays a major role in the pathogenesis of organ dysfunction and failure, PGRN might be an early predictor for mortality.

Numerous biomarkers have been evaluated for their ability to predict mortality across various cohorts of critically ill patients. However, most studies assess predictive correlations between biomarkers and mortality across entire cohorts, without stratifying by illness severity. In consequence, only a few biomarker studies have focused on critically ill patients with relatively low illness severity, as defined by lower Sequential Organ Failure Assessment (SOFA) scores [17,18]. More recently, this approach has been refined through the concept of burden of organ dysfunction (BOD), which incorporates SOFA assessments over time [19]. By evaluating the SOFA score every 8 h over a 5-day period, kinetic parameters, such as maximum SOFA, mean SOFA, and a normalized area score (NAS), calculated as the area under the SOFA curve divided by the observation period, were derived [19]. Moreover, a strong linear correlation was observed between the initial SOFA value and the NAS, which validates the initial SOFA score as a measure for the BOD defined by the NAS [19]. In this context, a SOFA score of 8 identified a critical threshold, marking an inflection point where mortality rates sharply increased. Consequently, patients with an initial SOFA score of 8 or lower were classified as having low BOD, while those with a score of 9 or higher were regarded as having high BOD, which was also supported by a statistically significant difference in NAS between the two groups [19].

Given the observed importance of the initial SOFA score as an early measure of disease burden [19], and inspired by the previous work by Baldirà et al. [17], we hypothesized that PGRN might serve as an early predictor of mortality in less severely ill patients. Specifically, we aimed to determine whether the PGRN levels could predict mortality in low BOD (initial SOFA ≤ 8) versus high BOD (initial SOFA > 8) patients. Additionally, we investigated correlations between PGRN and other biomarkers of inflammatory and hypoxic processes, seeking deeper insights into the clinically relevant pathophysiological conditions underlying its production.

## 2. Materials and Methods

### 2.1. Ethics and General Aspects

This study represents a secondary analysis of data obtained from a previous monocentric, prospective observational clinical study [20]. The protocol of the respective study was approved by the local ethics committee (registration number: 2016-643-N-MA, approved on 5 January 2017), as referenced in the Institutional Review Board Statement. Written informed consent was obtained from all participants or their legal representatives. Patients who were initially unable to provide consent were given the opportunity to withdraw their participation upon recovery.

This study adhered to the guidelines outlined in the Strengthening the Reporting of Observational Studies in Epidemiology (STROBE) statement (available at https://www.strobe-statement.org/ (accessed on 12 January 2026)). It was conducted in the 24-bed intensive care unit (ICU) of the Department of Anesthesiology, Surgical Intensive Care Medicine and Pain Medicine, Mannheim University Hospital, Mannheim Medical Faculty, University of Heidelberg. The recruitment period spanned from June 2017 to June 2019. Due to the timing of the original study’s initiation, patient enrollment followed the sepsis-1/2 criteria, which were the prevailing definitions at that time. Consequently, the inclusion criteria required patients to either exhibit a continuous systemic inflammatory response syndrome (SIRS) status for at least 48 h or meet the sepsis-1/2 definition for sepsis [21] upon ICU admission (for further details, see reference [20]). Patient status was evaluated at the time of study inclusion (denoted as time point 1) and subsequently every 8 h for up to five days, yielding a maximum of 15 assessment time points. The study period ended upon discharge from the ICU, which resulted in a total of 1272 assessment time points for the 99 included patients. At each assessment, the SOFA score was calculated, and blood samples were collected to measure the PGRN plasma concentrations. Clinical SOFA components, including hemodynamic and vasopressor requirement, level of consciousness, and PaO_2_/FiO_2_ ratio, were evaluated within each respective 8 h time window. Laboratory parameters required for the SOFA calculation (serum creatinine, total bilirubin, and platelet count) were obtained according to routine clinical laboratory schedules, typically once daily. If additional laboratory measurements were performed within the same 24 h period for clinical reasons, these values were incorporated into the corresponding SOFA assessment. Clinical data from the ICU patients were recorded by the IntelliSpace Critical Care and Anesthesia (ICCA)^TM^ system (Philips N.V., Amsterdam, The Netherlands). Patients meeting any of the following exclusion criteria were not included in this study: age < 18 years, immunosuppression, end-stage renal failure, pregnancy, ongoing extracorporeal membrane oxygenation (ECMO) therapy, or a primary neurosurgical diagnosis, as this could introduce confounding neuroinflammatory factors. Baseline and clinical characteristics of the study population have been described previously [19].

### 2.2. Measurement of Progranulin Levels

Venous blood plasma samples for the PGRN measurements were collected either through a central venous catheter or a peripheral indwelling cannula. To minimize bias, laboratory staff processing the samples were unaware of the patients’ clinical status and medical specialists involved in the patients’ care were blinded to the PGRN measurements obtained by an enzyme-linked immunosorbent assay (Progranulin ELISA, Product E103, Mediagnost, Reutlingen, Germany). Lactate concentrations were assessed using arterial blood gas analyses performed with a blood gas analyzer (Radiometer ABL 800 Flex, Radiometer, Willich, Germany). S-Adenosylhomocysteine (SAH), another marker for hypoxia, was measured as described recently [20]. To capture the cumulative biomarker exposure over time, we calculated the NAS, defined as the area under the concentration–time curve divided by the observation period. This approach is analogous to the concept of “normalized lactate load” [22] or “time-weighted average lactate” [23], previously described for lactate kinetics in critically ill patients [24,25]

### 2.3. Statistical Analysis

To characterize the study cohort, continuous variables were compared using *t*-tests (Satterthwaite), while categorical variables were analyzed using Chi^2^ tests. For non-normally distributed data, the Mann–Whitney U test was applied. Unless stated otherwise, continuous variables are presented as medians with interquartile ranges (IQRs) and categorical data are reported as absolute numbers and percentages.

Univariate logistic regression analyses were performed to evaluate associations between the predictors PGRN, SOFA, or C-reactive protein (CRP), including their kinetic applications and the response variable in-hospital mortality. Model performance was assessed via the estimated regression coefficient to the base *e* (2.7182), reflecting the factor by which the odds of in-hospital death change per one-unit increase in the respective variable. To assess the prognostic capacities of the individual parameters, especially their ability to distinguish between survivors and non-survivors, receiver operating characteristic (ROC) curves were generated and the area under the ROC curve (AUROC) values were calculated. Differences in the AUROC values were evaluated both within and across groups and tested for statistical significance. The semi-quantitative interpretation of the AUROC values followed the criteria proposed by Hosmer and Lemeshow [26]. For the analyses of correlations of the initial PGRN values with the initial values of SOFA, CRP, INR, platelet count, lactate, and S-adenosylhomocysteine, Spearman’s rank and linear regression correlation coefficients were calculated. Effect sizes of the correlations were classified according to Cohen’s guidelines [27].

Despite predefined hypotheses, all statistical tests were conducted two-sided and a *p*-value < 0.05 was considered statistically significant. For multiple comparisons between two groups, the level of significance was adjusted using the Bonferroni correction method [28] as indicated. All statistical analyses were performed using SAS software Version 9.4 (SAS Institute, Cary, NC, USA) and IBM SPSS Statistics Version 27 (IBM, Albany, NY, USA).

## 3. Results

### 3.1. Categorizing the Patients Based on the Median Initial SOFA Score

In a previous study [19], 99 critically ill patients were enrolled and retrospectively categorized into low and high BOD groups based on the total cohort’s initial median SOFA score of 8 used as a classifier. This cutoff was not only the median SOFA score of the cohort at study inclusion but furthermore it did not result in a statistically significant difference in mortality between the two groups. Consequently, this approach was chosen to show that differences observed between patients with low and high BOD are likely due to differences in the severity of ongoing pathogenetic mechanisms before these differences are reflected in mortality rates. Additionally, an inflection point in the mortality rate between initial SOFA scores of 8 and 9 supported this cutoff (for further details see [19]). The strong correlation of individual initial SOFA values with the corresponding BOD values, assessed by the individuals’ subsequent five days NAS of SOFA (*r* = 0.813), supported the use of the median initial SOFA score as the cutoff value for predictive grouping of patients to either belong to the low or high BOD group, respectively. Accordingly, 53 patients with an initial SOFA ≤ 8 were assigned to the low BOD group and 46 with SOFA > 8 were assigned to the high BOD group.

### 3.2. Baseline and Clinical Characteristics of the Study Participants

At study inclusion, comprehensive baseline data were obtained for all patients, encompassing demographics, primary diagnosis, comorbidities, clinical course, vital signs, laboratory parameters (clinical chemistry and hematology), clinical scores, and hypoxia associated biomarkers. Comparative analyses were performed between patients with low and high BOD, as well as between survivors and non-survivors within each group.

Despite comparable mortality rates in patients with low and high BOD, the overall clinical characteristics differed markedly between the two groups. Individuals with high BOD were younger and more often male, and they required mechanical ventilation and vasopressor therapy more frequently. They also presented with a higher shock index and experienced longer ICU stays. Traumatic etiologies, particularly polytrauma and major bleeding, were more common among patients with high BOD, whereas sepsis and major surgical interventions occurred less frequently. Conversely, chronic conditions, such as cardiac or pulmonary disease, arterial hypertension, and diabetes mellitus, were less prevalent in this group.

Biochemical indicators of organ dysfunction were consistently higher in patients with high BOD, including markers reflecting the impairment of renal, hepatic, and pancreatic functions. Parameters associated with inflammation and infection were also more strongly altered, pointing toward a more pronounced inflammatory response or increased activation of coagulatory pathways. This greater disease severity was further reflected by significantly higher requirements for sedation and therapeutic interventions, as indicated by lowered RASS and elevated TISS scores. In parallel, the SOFA scores were approximately twice as high in the high BOD group. The baseline plasma levels of lactate were likewise significantly increased in these patients (*p* = 0.004, respectively; Table 1).

Within the low BOD subgroup, survivors and non-survivors showed largely comparable characteristics, with the exception of the SOFA score, which was higher among the non-survivors. By contrast, more pronounced differences were observed within the high BOD cohort. Here, non-survivors tended to be older, had shorter ICU stays, and were more frequently admitted with sepsis, whereas polytrauma was less common. The markers of renal dysfunction, including creatinine and urea, were significantly higher in non-survivors, who also exhibited greater illness severity, as reflected by elevated TISS, SAPS II, and SOFA scores. Furthermore, initial plasma concentrations of lactate were significantly higher in non-survivors compared with survivors (*p* = 0.031, respectively; Table 1).

### 3.3. Time Course of Progranulin Plasma Concentrations in Patients Grouped by BOD and Mortality

**Figure 1:**
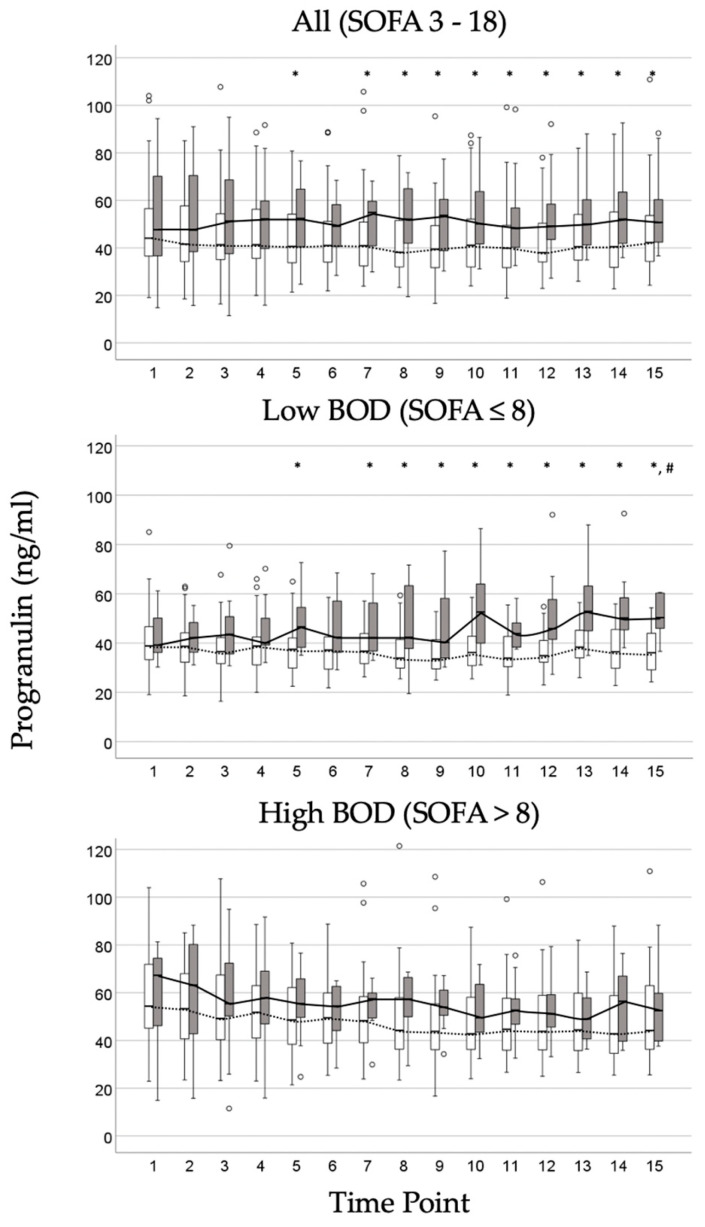
Progression of progranulin plasma concentrations in survivors and non-survivors grouped by their burden of organ dysfunction (BOD). The upper, middle, and lower panels show time series data for all patients with initial SOFA scores ranging between 3 and 18, and after classification in those with a low (initial SOFA ≤ 8) and high BOD (initial SOFA > 8), respectively. The data for survivors and non-survivors are represented by white and grey boxplots, where the top and bottom lines of the box denote the 25th and 75th percentiles, respectively. The lower and upper whiskers represent the 10th and 90th percentiles. The median value is indicated by the horizontal line within the box. A line connecting these median values (dotted for survivors, solid for non-survivors) illustrates the areas under the curves of each contributing patient. Outliers, which are values greater than 1.5 standard deviations from the mean of the variable, are marked with empty circles. Comparisons between survivors and non-survivors were made at each time point using the Mann–Whitney U test (* *p* ≤ 0.05) and with significance levels adjusted according to the Bonferroni correction (# *p* ≤ 0.0033). For all patients and for patients with low BOD, statistically significant differences were observed in progranulin plasma concentrations. However, when levels of statistical significance were corrected according to Bonferroni, only in patients with low BOD differences between survivors and non-survivors remained significant at the last time point of measurement. Patients with high BOD showed no significant differences at all.

A total of 1272 assessment time points were analyzed in 99 patients during the study period. Due to incomplete datasets, 1257 PGRN plasma samples (98.8%) were included in the analysis. These serial measurements served for calculating the kinetic parameters as previously described [19]. Besides the BOD group membership, patients were stratified according to survival status at each time point (see Figure 1). In the total cohort, the PGRN levels were significantly higher in non-survivors compared to survivors (see upper panel Figure 1). This difference was driven by a statistical significance within the subgroup of patients with low BOD (see middle panel Figure 1). By contrast, no significant differences were observed between survivors and non-survivors in the high BOD subgroup at any time point (see lower panel Figure 1). After adjustment for multiple comparisons using the Bonferroni correction, statistical significance persisted only in the low BOD group and at the final time point of measurement.

### 3.4. Performance of Progranulin Plasma Concentrations in Patients with Low and High BOD for Prediction of Mortality

A comparison of the initial values and kinetic parameters of the serially measured plasma PGRN concentrations revealed significant differences between survivors and non-survivors in the total study cohort for the maximum, mean, and NAS (Table 2). When stratified by low BOD and high BOD, significant differences between survivors and non-survivors were found solely in the low BOD group for the maximum, mean, and NAS. By contrast, no statistically significant differences in the plasma PGRN levels were detected in the high BOD group (Table 2).

Univariate logistic regression analyses were performed to assess the predictive value of the initial and kinetic PGRN plasma concentrations for in-hospital mortality, both in the total cohort and stratified by BOD. In the total cohort, the maximum PGRN levels, mean, and NAS were significantly associated with in-hospital mortality, with odds ratios (OR) of 1.024 (95% CI: 1.001–1.048; *p* = 0.045), 1.035 (95% CI: 1.002–1.069; *p* = 0.037), and 1.040 (95% CI: 1.007–1.074; *p* = 0.017), respectively. The corresponding AUROC values were 0.667, 0.663, and 0.673, all of which were statistically significant, suggesting an overall moderate discriminatory power of the kinetic parameters. By contrast, the initial PGRN concentration showed no significant association with in-hospital mortality in the total cohort (OR 1.010 (95% CI: 0.985–1.035; *p* = 0.443), AUROC 0.544) (Table 3).

**Figure 2:**
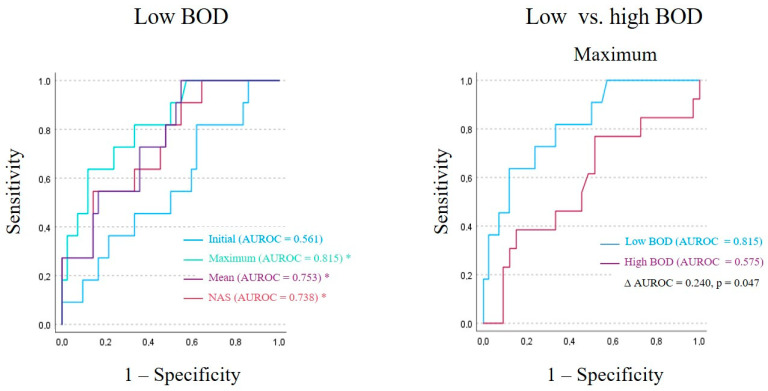
Receiver operating characteristic (ROC) curves for mortality prediction based on univariate logistic regression models using initial and kinetic parameters of progranulin plasma concentrations in patients with low burden of organ dysfunction (BOD) and comparison between low and high BOD patient groups for maximum progranulin values. In low BOD patients (left panel), the AUROC values of kinetic parameters were all significantly higher compared to the initial progranulin value (* *p* ≤ 0.01), with the highest AUROC for progranulin maximum values. A comparison of the AUROC values for kinetic parameter between low and high BOD groups revealed a statistically significant difference for the parameter maximum PGRN, with a positive Δ AUROC (Δ AUROC = AUROC _low BOD_ − AUROC _high BOD_) supporting improved mortality discrimination in the low BOD cohort. Abbreviations: AUROC = area under the receiver operator characteristic curve.

When stratified by BOD, the predictive capacity of PGRN was more pronounced in patients with low BOD. In this subgroup, maximum values, mean, and NAS were significantly associated with in-hospital mortality, yielding odds ratios of 1.075 (95% CI: 1.022–1.131; *p* = 0.005), 1.102 (95% CI: 1.025–1.184; *p* = 0.008), and 1.093 (95% CI: 1.021–1.170; *p* = 0.011), respectively. The AUROC values for these parameters were all > 0.7 and statistically significant (maximum: 0.815, *p* = 0.001; mean: 0.753, *p* = 0.010; NAS: 0.738, *p* = 0.016), indicating excellent and acceptable levels of discrimination according to the classification by Hosmer and Lemeshow [26]. The initial PGRN concentration again failed to predict in-hospital mortality in this subgroup (OR 1.024 (95% CI: 0.980–1.069; *p* = 0.292); AUROC: 0.561, *p* = 0.097) (Table 3 and Figure 2).

No statistically significant associations were observed in the high BOD group, in which neither the initial nor the kinetic PGRN parameters showed predictive values for in-hospital mortality. The AUROC values in this group remained consistently below 0.6 (Table 3).

A comparison of the AUROC values between the low and high BOD subgroups (∆ AUROC) revealed a statistically significant difference for the maximum PGRN concentrations (∆ AUROC = 0.245; *p* = 0.047), suggesting that the predictive utility of this parameter is particularly pronounced in patients with low BOD. No significant intergroup differences in the AUROC values were found for initial values, mean, or NAS (Table 3 and Figure 2).

By contrast, in patients with low BOD, the SOFA score metrics, except for the initial value, were not significantly associated with in-hospital mortality (see Supplementary Table S1). As expected in the high BOD group, all SOFA metrics were significantly predictive. Nonetheless, no significant differences were obtained in the AUROC comparisons for all SOFA metrics between the low and high BOD groups.

When CRP was evaluated for its ability to predict mortality, none of the parameters reached statistical significance in the low BOD group (see Supplementary Table S2). In the high BOD group, only CRP NAS showed a statistically significant association. As compared to their initial values, the AUROC values of the kinetic parameters increased steadily but remained within the range of poor discrimination. Overall, these results suggest that the PGRN mean, NAS, and especially the maximum levels outperformed the corresponding SOFA and CRP metrics in predicting mortality in the low BOD group.

To assess the robustness of PGRN’s predictive value, a sensitivity analysis was performed by varying the SOFA cutoff (Table 4). When the SOFA cutoff was lowered from 8 to 6, the overall pattern observed for the cutoff of 8 was preserved (compare Table 3 and Table 4). In the SOFA ≤ 6 group, the predictive performance of kinetic parameters increased further, accompanied by AUROC values rising into the excellent range. This effect was most pronounced for the maximum PGRN values. In the SOFA > 6 group, none of the parameters reached statistical significance, and AUROC values remained in the poor range, again with the exception of the maximum PGRN. As observed previously for a SOFA cutoff of 8, the difference in the AUROC values between SOFA ≤ 6 and >6 was large and statistically significant.

When applying a SOFA cutoff of 10, mortality prediction did not reach statistical significance for any parameter except for the maximum PGRN in the SOFA ≤ 10 group, exhibiting an AUROC in the acceptable range. The differences in the AUROC values between SOFA ≤ 10 and >10 were not statistical significance for any parameter.

A decrease in the SOFA cutoff from 10 to 6 was inversely associated with increasing AUROC values for maximum PGRN resulting in the following rank order: SOFA ≤ 10 (0.691), <SOFA ≤ 8 (0.815), <SOFA ≤ 6 (0.854). This observation indicates that the predictive performance of mortality by the maximum PGRN improved progressively in patients with lower degrees of organ dysfunction.

### 3.5. Cutoff Values for Mortality Prediction by Progranulin Kinetic Parameters

The cutoff values for mortality prediction by progranulin kinetic parameters were determined in the patient group with low BOD (SOFA ≤ 8). The optimal cutoff for the maximum PGRN concentration for the detection of non-survivors was 47.5 ng/mL, corresponding to a sensitivity of 81.8% and a specificity of 66.7% (Youden index = 0.485). When AUROC curves of the PGRN mean and NAS were used to determine the cutoff values yielding the same sensitivity of 81.8%, the resulting specificities were 47.6% and 45.2% for cutoff values of 36.1 ng/mL and 35.4 ng/mL, respectively. At a sensitivity of 100%, i.e., detecting all non-survivors, specificities decreased to 40.5%, 45.2%, and 35.7% for maximum, mean, and NAS values with corresponding cutoff values of 41.2 ng/mL, 35.5 ng/mL, and 33.0 ng/mL.

### 3.6. Frequency Distribution of Maximum Progranulin Values During the Study Period and Rates of Increase in Progranulin Concentrations in Patients with Low BOD (SOFA ≤ 8)

Histograms of the maximum PGRN values determined across time points 1 to 15 of the study period showed a positively (right-)skewed distribution in survivors and a negatively (left-)skewed distribution in non-survivors (see Supplementary Figure S1). Among survivors, 64% reached the maximum PGRN concentration within the first 5 time points, whereas 64% of non-survivors reached their peak between time points 10 and 15. This difference in frequency distributions was statistically significant. Despite this, substantial overlap between the distributions remained, making it challenging to determine how the study period could be shortened, sampling intervals extended, or sampling frequency reduced without risking missed detection of true maximum concentrations. However, when univariate logistic regression was applied to data obtained with longer sampling intervals, i.e., every 24 h instead of every 8 h, maximum PGRN values still demonstrated acceptable predictive performance (AUROC 0.746 for 24 h vs. 0.815, for 8 h intervals). Of note, rates of increase in the PGRN concentrations were predictive of mortality only when calculated from values obtained between time point 1 and the individual maximum (AUROC (SE) 0.736 (0.087), *p* = 0.017; regression coefficient (mean, SE): 0.510, 0.232; odds ratio (95% CI): 1.665 (1.057–2.622), *p* = 0.028). When rates of increase in the PGRN concentrations were calculated using shorter intervals, i.e., from time point 1 to time points 3, 5, or 8, they failed to predict mortality.

### 3.7. Time Between Progranulin Maximum and Death

**Figure 3:**
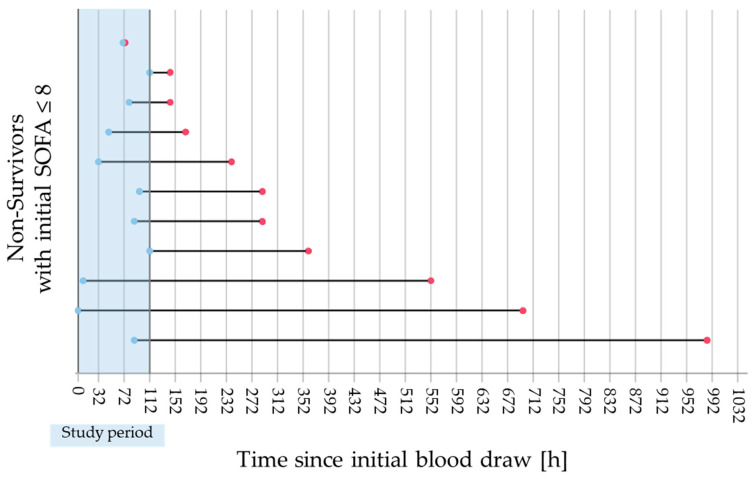
Time between maximum progranulin levels and death. In the subgroup of patients with low BOD, 11 individuals died, each represented in the graph by a horizontal line. Maximum levels of progranulin are indicated by blue dots within the study period (highlighted in azure), while time points of death are indicated by red dots. Time 0 h, 32 h, 72 h, and 112 h refers to time points 1, 5, 10, and 15 of the study period. The time between peak progranulin levels and death ranged from 32 to 896 h.

To serve as a clinically useful predictor of imminent death, a biomarker should enable timely diagnostic and therapeutic interventions. As illustrated in Figure 3, the maximum values of PGRN measured during the study period in patients with low BOD occurred prior to death in all cases except one. In that exceptional case, the patient developed sudden onset of mesenteric ischemia and died on the same day. In the remaining cases, the number of blood sampling intervals between the time point of the maximum PGRN levels and death ranged from 4 to 112. Given that each interval corresponds to 8 h, this translates to a minimum window of 32 h for potential clinical intervention before death occurred.

### 3.8. Correlation Between Values of Progranulin and SOFA, Inflammatory and Coagulation Parameters, as Well as the Hypoxia Biomarker S-Adenosylhomocysteine

**Figure 4:**
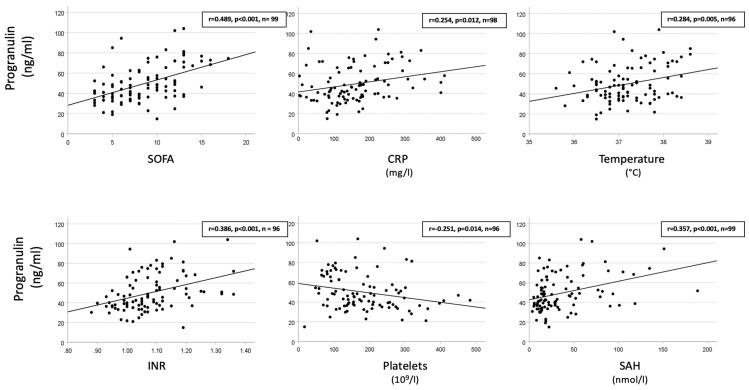
Correlations between initial values of progranulin and SOFA, inflammatory and coagulatory parameters, as well as the hypoxia marker S-adenosylhomocysteine in the total cohort of critically ill patients. Each data point represents a pair of variables from an individual patient. Linear regression coefficients calculated by Pearson (r) and *p*-values are given. Based on Cohen’s thresholds for evaluating correlation effect sizes [27], progranulin showed medium-sized correlations with SOFA, INR, and SAH, while its correlations with CRP, temperature, and platelet counts fell within the small effect-size range. Abbreviations: SOFA = Sequential Organ Failure Assessment; CRP = C-reactive protein; INR = international normalized ratio; SAH = S-adenosylhomocysteine.

To explore potential pathophysiological links between PGRN and critical illness, correlations between the initial PGRN concentrations and key clinical and biochemical markers were assessed across the total cohort of critically ill patients. As shown in Figure 4, statistically significant positive correlations were observed between the initial PGRN levels and several markers of organ dysfunction, inflammation, coagulation, and tissue hypoxia.

Specifically, the PGRN concentrations correlated with the SOFA score (*r* = 0.489; *p* = 0.014) with medium effect size according to Cohen [27], indicating a potential association with the extent of global organ dysfunction at baseline. Among inflammatory markers, PGRN showed a weak but significant correlation with CRP (*r* = 0.254; *p* = 0.012, small effect size) and body temperature (*r* = 0.284; *p* = 0.005, small effect size), suggesting responsiveness to systemic inflammation.

Regarding coagulation parameters, the PGRN levels correlated positively with the international normalized ratio (INR; *r* = 0.386; *p* < 0.0014, medium effect size) and inversely with platelet counts (*r* = −0.251; *p* = 0.014, small effect size), both reflecting the coagulation pathway activation commonly observed in critically ill patients. Moreover, a significant correlation was found with S-adenosylhomocysteine (SAH; *r* = 0.357; *p* < 0.001, medium effect size), a biomarker linked to hypoxia and impaired methylation, underscoring a potential link between PGRN and hypoxic stress responses. The association of PGRN with hypoxic stress was also supported by its positive correlation with lactate plasma concentrations (*r* = 0.354, *p* < 0.001).

In the group of low BOD patients, the PGRN maximum values were significantly associated with the maximum values of SOFA, CRP, INR, platelets, and hypoxia parameters (see Supplementary Table S3). In contrast to the PGRN peak levels, the maximum SOFA values did not reach any significant correlation with the respective parameters in the low BOD group.

Collectively, these findings suggest that elevated PGRN levels may already at baseline reflect a composite signal of organ dysfunction, systemic inflammation, coagulopathy, and hypoxic stress in critically ill patients. These results were confirmed for correlations of maximum values in the subgroup of patients with low BOD. Moreover, respective correlations of the maximum PGRN values outperformed those of the maximum SOFA values.

## 4. Discussion

### 4.1. Mortality Prediction by PGRN Stratified by Severity of Organ Dysfunction

In critically ill patients, the SOFA score is a well-established tool to assess and monitor the severity of organ dysfunction irrespective of whether the underlying etiology is non-infectious or infectious [29]. The onset of organ dysfunction serves as a critical warning sign of ongoing pathological processes, prompting clinicians to intensify diagnostic and therapeutic efforts. Timely clinical attention can facilitate accurate diagnosis and may allow for mitigation—or even reversal—of the underlying pathology. Consequently, scoring systems, such as the SOFA score, are widely used to quantify organ dysfunction and to predict its progression toward multiple organ failure and death. Its prognostic accuracy is generally highest in ICU patients with pronounced organ failure [30]. However, in less severely ill patients, SOFA may be less reliable [31,32], potentially overlooking subtle signs of deterioration that are not captured by organ failure metrics alone.

In the present study of a cohort of critically ill patients with higher PGRN levels, particularly dynamic indices/kinetic parameters, such as maximum, mean, and NAS measured during the first five days following ICU admission, were associated with increased in-hospital mortality. Notably, the initial PGRN concentrations at ICU admission did not differ significantly between survivors and non-survivors, whereas the maximum PGRN levels were markedly elevated in those who died. Time-integrated PGRN measurements (maximum, mean, and NAS) showed significant prognostic value, especially in the low BOD patients (AUROC values 0.815, 0.753, and 0.738; *p* = 0.001, 0.010, and 0.016, respectively). By contrast, in patients with high BOD, neither initial nor serial PGRN metrics predicted outcome (all AUROC values ~0.5–0.6, *p* > 0.257). This divergence was further supported by a significant difference in prognostic performance between the low and high BOD groups for maximum PGRN (Δ AUROC 0.240, *p* = 0.047). Notably, in patients with low BOD, maximum levels enabled prediction of death at least 32 h in advance. By contrast, neither SOFA nor CRP kinetic parameters were associated with mortality, despite the elevated risk indicated by PGRN parameters in this subgroup of less severely ill patients. Sensitivity analyses using higher and lower SOFA cutoffs (10 and 6, respectively) confirmed the robustness of the predictive value of PGRN kinetic parameters. This was particularly evident for the maximum PGRN levels, as their association with mortality and their AUROC values further increased in patients with a lower burden of organ dysfunction when the SOFA cutoff was reduced.

Our findings align with the emerging evidence identifying PGRN as a prognostic biomarker in critical ill patients. Liu et al. determined the serum levels of PGRN in patients with candidemia and evaluated their association with mortality [12]. In their prospective, multicenter study conducted in ICUs at two university hospitals, in two cohorts of candidemic patients (consisting of a discovery cohort, *n* = 62 and a validation cohort, *n* = 70), the serum PGRN levels were significantly associated with mortality, demonstrating excellent discriminatory performance with AUC values of 0.892 (95% CI, 0.787–0.956, *p* < 0.0001, discovery cohort) and 0.843 (95% CI, 1.0041–1.0220, *p* < 0.0001, validation cohort). The SOFA scores reported in Liu’s study (median 4, IQR 2–7)) were comparable to those observed in our low BOD group (6, IQR 4–7) further supporting the relevance of PGRN as a prognostic marker in patients with moderate disease severity. Shan et al. [33] identified PGRN as an independent predictor of 28-day mortality, reporting an AUC of 0.872 in a multivariate Cox regression analysis. The mean SOFA score in their cohort (mean ± SD: 7.12 ± 4.10, *n* = 128) was again comparable to that observed in our low BOD group. By contrast, Brandes et al. [34] observed in a large ICU study that mortality prediction in critically ill ICU patients by plasma PGRN concentrations was poor (AUC 0.63 (95% CI 0.54–0.72, *n* = 241). Interestingly, the severity of illness in their cohort was substantially higher, with median SOFA scores of 13.0 (IQR: 10.0–15.0) in the exploratory (*n* = 114) and 12.0 (IQR: 8.0–14.0) in the confirmatory cohort (*n* = 127)—ranges that closely match those in our high BOD group (median: 12, IQR: 10–13). Our results support the notion that the prognostic utility of PGRN is most pronounced in patients who have not yet progressed to advanced organ failure. In patients who already have high SOFA scores, PGRN levels may be uniformly elevated among both survivors and non-survivors, as observed in our high BOD group, where even kinetic PGRN parameters failed to show statistically significant differences and thus lacked discriminative power.

Taken together with our findings, the previous studies support the role of PGRN as a predictive biomarker of mortality in critical illness. Importantly, our data extend this concept by showing that, in patients with low BOD, a subgroup typically associated with favorable outcomes, serial (NAS and mean) or maximum PGRN offer valuable prognostic information. In this ostensibly low-risk population, non-survivors exhibited significantly higher maximum PGRN levels (62.1 ng/mL) compared to survivors (43.0 ng/mL), despite similar baseline values. This underscores the superior predictive value for mortality by kinetic parameters [35]. Thus, kinetic parameters of serial PGRN measurements may be particularly useful in detecting subtle deteriorations in patients who are not otherwise flagged as high-risk by organ dysfunction scores such as SOFA.

### 4.2. PGRN and Sequence of Events in the Pathogenesis of Organ Dysfunction

Intensive care specialists widely agree that damage to the microcirculation, most commonly caused by inflammatory processes, impairs blood flow-dependent oxygen supply. Inevitably, this will result in tissue hypoxia, which in turn leads to cellular and organ dysfunction [36]. However, only a few studies have investigated the microcirculatory biomarkers which reflect endothelial responses to hypoxic stress. One such candidate is adrenomedullin, a molecule induced by hypoxia inducible factor (HIF) [37], which can be monitored via its stable degradation product, mid-regional pro-adrenomedullin (MR-proADM) [38]. Assuming that hypoxia represents an early event in the pathogenesis of organ dysfunction, MR-proADM was evaluated for its prognostic utility in patients with SOFA scores ≤ 6 [17,39] or ≤7 [40] to test its predictive power under more difficult conditions, namely in less severely ill patients. Specifically, in a pioneering study by Baldirà et al. [17], MR-proADM demonstrated good prognostic accuracy (AUROC 0.70) in sepsis patients with low SOFA scores (≤6), a concept analogous to our low BOD cohort. Similar to MR-proADM, progranulin is also upregulated during hypoxia [13,14,15,16], and it exerts anti-inflammatory, immunosuppressive effects [41,42]. Moreover, PGRN is produced in response to microcirculatory failure due to non-infectious [16,43] and infectious inflammatory conditions, for instance those elicited by LPS [41] or sepsis [33]. Assuming that PGRN is upregulated by hypoxia secondary to microcirculatory failure, it is reasonable to expect PGRN to correlate with the clinical parameters of inflammation, disseminated coagulation, and hypoxia-related metabolites. Such correlations have been observed in several clinical studies [12,33] and have been confirmed by our results. Specifically, in our total cohort, the initial PGRN levels correlated with SOFA, CRP, INR, platelet count, lactate, and S-adenosylhomocysteine, a recently described marker of hypoxia [19,20,35]. According to Cohen’s guidelines [27], the correlations of progranulin with SOFA, INR, and SAH fall within the range of medium effect sizes, whereas the correlations with CRP, temperature, and platelets represent small effect sizes. These correlations were also observed for the maximum PGRN values in the low BOD group. By contrast, maximum SOFA scores in the same patient group did not show significant correlations with these parameters, underscoring the added value of PGRN in capturing early pathophysiological changes not reflected by conventional organ dysfunction metrics.

Thus, the early increase of plasma concentrations of PGRN in non-survivors, compared to survivors, might reflect differences in the intensity of the pathophysiological processes contributing to the microvascular damage subsequently leading to hypoxia. Subtle variations in hypoxic burden might be captured by the upregulation of PGRN well before overt organ dysfunction becomes clinically apparent. In fact, in our low BOD patients, we found no significant correlation between the maximum levels of PGRN and those of the hypoxia biomarker S-adenosylhomocysteine in survivors (*r* = 0.156, *N* = 42, *p* = 0.325), whereas a strong and statistically significant correlation was observed in non-survivors (*r* = 0.645, *N* = 11, *p* = 0.032). To date, no canonical hypoxia responsive element (HRE) has been conclusively identified in the promoter region of the human progranulin (PGRN) gene. Given that PGRN protein expression is upregulated under hypoxic conditions [13,14,15,16], this area warrants further investigation to elucidate the molecular mechanisms underlying PGRN regulation in hypoxia-driven organ dysfunction.

### 4.3. Clinical Implications

From a clinical perspective, our study suggests that serial monitoring of PGRN may help identify ICU patients at “hidden” risk of unfavorable outcome. Particularly among patients presenting with low BOD according to established scores of organ dysfunction, rising PGRN to peak concentrations might indicate an occult trajectory towards deterioration. In our cohort, the optimal cutoff for the maximum PGRN concentration for the detection of non-survivors in patients with low BOD (SOFA ≤ 8) was 47.5 ng/mL, corresponding to a sensitivity of 81.8% and a specificity of 66.7%.

Although the finding that maximum PGRN levels predicted death at least 32 h in advance is valid in retrospect, it is difficult to apply prospectively. Clinicians cannot know that a given value represents the ‘maximum’ until levels subsequently decline, or the patient dies. This creates a need for predictive value in the rate at which PGRN levels rise. However, except for the rate of increase toward the maximum, these kinetic parameters did not show predictive utility. Consequently, our data suggest repeated measurements of PGRN plasma concentrations to avoid missing the true peak. Encouragingly, extending the sampling interval from 8 to 24 h during the first five days of monitoring still maintained mortality-prediction performance within an acceptable range, thereby reducing workload. Taken together, our findings support the notion that PGRN likely represents a predictive biomarker, particularly in the early or deceptively benign phases of critical illness. Its ability to reflect both the intensity and progression of occult pathological processes, before overt organ dysfunctions become apparent, makes PGRN a promising candidate for early risk stratification and clinical monitoring, particularly in less severely ill ICU patients.

Importantly, the present findings should not be interpreted as evidence that PGRN itself represents a direct therapeutic target in critical illness. Rather, elevated PGRN likely reflects underlying biological stress responses, such as inflammation, tissue injury, and dysregulated host response. Nevertheless, emerging translational research suggests that PGRN levels can be pharmacologically modulated. For example, the monoclonal antibody latozinemab, which targets the sortilin–PGRN axis, has been shown in a phase-1 clinical study to substantially increase plasma and cerebrospinal fluid PGRN concentrations in patients with progranulin-associated frontotemporal dementia, demonstrating proof-of-mechanism for the therapeutic modulation of PGRN biology [44].

While such approaches are currently unrelated to critical care medicine, and no interventional ICU studies targeting PGRN exist to date, these findings highlight that PGRN is not merely an epiphenomenal biomarker but part of the biologically active signaling pathways that may become therapeutically relevant in the future.

Overall, these results support further investigations into PGRN as a tool for early risk stratification in critical illnesses.

### 4.4. Study Limitations

This study has several limitations. First, it was a single-center study with a modest sample size (*n* = 99), which may limit the generalizability of our findings. Patient populations at individual centers may reflect specific referral patterns, case mixes, and local treatment practices. Therefore, the underlying causes of critical illness and indications for ICU admission may differ across institutions. This issue was further compounded by the formation of subgroups, which further diminished the number of patients available for statistical comparisons. Nonetheless, both inter- and intragroup comparisons were conducted on a population delineated by prospectively defined inclusion and exclusion criteria. Although a larger sample could enhance statistical power, biomarkers were repetitively monitored at 8 h intervals over up to five days. Depending on the question of interest, if this longitudinal approach was to be shifted to a horizontal cross-sectional design focusing on the time point of admission, the requisite patient count would be 1272, corresponding with a total of 1272 assessment-time points due to the longitudinal study design. Second, we defined low versus high BOD using an initial SOFA score cutoff of 8, based on prior data from this cohort; while this stratification revealed distinct prognostic patterns, it may not be universally applicable and should be validated in larger, multi-center studies. Third, while PGRN outperformed SOFA and other biomarkers (e.g., CRP), it is not yet available as a routine clinical test. Fourth, the statistical analyses were primarily based on univariate logistic regression models. While this approach was chosen to assess the independent prognostic signal of PGRN parameters, it does not fully account for potential confounding effects from clinical variables, comorbidities, or concurrent therapies. Future studies incorporating multivariate models are therefore required to confirm the independent prognostic value of PGRN.

### 4.5. Future Perspectives

The present study provides evidence that PGRN kinetics may serve as a promising biomarker for mortality prediction in critically ill patients with low BOD, a population in which early risk stratification remains challenging. In this cohort, dynamic PGRN parameters outperformed SOFA or CRP in the prediction of death. Specifically, PGRN maximum values performed best and identified deterioration well before death occurred, suggesting that serial PGRN measurements may capture the pathophysiological processes not reflected by conventional organ dysfunction scores.

However, the present study represents a pilot investigation providing initial evidence for the prognostic relevance of PGRN kinetics in this clinical setting, and establishes a rationale for validation in larger, multicenter cohorts to determine the robustness and generalizability of these findings across diverse ICU populations and clinical settings.

## 5. Conclusions

In conclusion, PGRN emerges as a promising prognostic biomarker in critically ill patients, especially for early identification of those at high mortality risk despite an initially low burden of organ dysfunction. Further prospective studies are needed to validate the utility of PGRN-guided risk stratification and to investigate whether interventions based on PGRN dynamics could improve outcomes particularly in less severely ill ICU patients.

## Figures and Tables

**Table 1:** Baseline and clinical characteristics of the study population.

								Low BOD (SOFA ≤ 8)					High BOD (SOFA > 8)				
		All (*N* = 99)	Low BOD (*N* = 53)		High BOD (*N* = 46)			Survivors (S) (*N* = 42)		Non-Survivors (NS) (*N* = 11)			Survivors (S) (*N* =33)		Non-Survivors (NS) (*N* = 13)		
	*n*		*n*		*n*		Low vs. High BOD	*n*		*n*		Low BOD S vs. NS	*n*		*n*		High BOD S vs. NS
**Demographics**																	
Age (years)	99	63 (53–76)	53	67 (60–78)	46	56 (49–68)	**0.015**	42	67 (60–78)	11	76 (61–80)	0.195	33	54 (43–63)	13	68 (59–78)	**0.001**
Male (%)		65 (66)		29 (54.7)		36 (78.3)	**0.014**		21 (50.0)		8 (72.7)	0.308		25 (75.8)		11 (84.6)	0.700
**Clinical course**																	
Mechanical ventilation (%)		86 (86.9)		41 (77.4)		45 (97.8)	**0.004**		31 (73.8)		10 (90.9)	0.420		33 (100)		12 (92.3)	0.283
Vasopressor therapy (%)		68 (69)		24 (45.3)		44 (95.7)	**<0.001**		17 (40.5)		7 (63.6)	0.190		31 (93.9)		13 (100)	1.000
ICU-LOS (days)	99	25 (16–47)	53	21.3 (14.2–37.9)	46	30.7 (20.4–68.9)	**0.006**	42	23.0 (14.9–38.0)	11	18.6 (10.3–32.3)	0.232	33	36.9 (21.7–86.8)	13	23.9 (14.7–35.9)	**0.048**
In-hospital mortality (%)		24 (24)		11 (20.8)		13 (28.3)	0.385										
**Primary diagnosis (%)**																	
Major surgery		11 (11.1)		9 (17.0)		2 (4.35)	**0.046**		7 (16.7)		2 (18.2)	1.000		1 (3.0)		1 (7.7)	0.489
Sepsis		20 (20.2)		15 (28.3)		5 (10.9)	**0.031**		11 (26.2)		4 (36.4)	0.708		1 (3.0)		4 (30.8)	**0.018**
Cardiac arrest		2 (2.0)		1 (1.9)		1 (2.2)	1.000		1 (2.4)			1.000		1 (3.0)			1.000
Polytrauma		42 (42.4)		16 (30.2)		26 (56.5)	**0.008**		13 (31.0)		3 (27.3)	1.000		23 (69.7)		3 (23.1)	**0.004**
Major bleeding		14 (14.1)		4 (7.6)		10 (21.7)	**0.043**		4 (9.5)			0.569		5 (15.2)		5 (38.5)	0.117
Respiratory insuff./ARDS		10 (10.1)		8 (15.1)		2 (4.4)	0.100		6 (14.3)		2 (18.2)	0.665		2 (6.1)			1.000
**Comorbidities (%)**																	
Cardiac		35 (35.4)		24 (45.3)		11 (23.9)	**0.027**		18 (42.9)		6 (54.5)	0.518		7 (21.2)		4 (30.8)	0.702
Vascular		21 (21.2)		12 (22.6)		9 (19.6)	0.709		7 (16.7)		5 (45.5)	0.098		4 (12.1)		5 (38.5)	0.092
Arterial hypertension		51 (51.5)		34 (64.2)		17 (37.0)	**0.007**		26 (61.9)		8 (72.7)	0.726		10 (30.3)		7 (53.8)	0.181
Pulmonary		12 (12.1)		10 (18.9)		2 (4.4)	**0.027**		6 (14.3)		4 (36.4)	0.187		2 (6.1)			1.000
Renal		20 (20.2)		13 (24.5)		7 (15.2)	0.250		8 (19.0)		5 (45.5)	0.112		4 (12.1)		3 (23.1)	0.385
Hepatic		6 (6.1)		2 (3.77)		4 (8.7)	0.412		1 (2.4)		1 (9.1)	0.375		3 (9.1)		1 (7.7)	1.000
Diabetes mellitus		17 (17.2)		13 (24.5)		4 (8.7)	**0.037**		10 (23.8)		3 (27.3)	1.000		3 (9.1)		1 (7.7)	1.000
Metabolic		10 (10.1)		3 (5.7)		7 (15.2)	0.181		2 (4.8)		1 (9.1)	0.510		6 (18.2)		1 (7.7)	0.654
Cerebral		11 (11.1)		7 (13.2)		4 (8.7)	0.476		6 (14.3)		1 (9.1)	1.000		3 (9.1)		1 (7.7)	1.000
Smoking		7 (7.1)		4 (7.6)		3 (6.5)	1.000		2 (4.8)		2 (18.2)	0.186		2 (6.1)		1 (7.7)	1.000
Alcoholism		6 (6.1)		2 (3.8)		4 (8.7)	0.412				2 (18.2)	0.040		3 (9.1)		1 (7.7)	1.000
**Clinical chemistry**																	
Creatinine (mg/dL)	96	0.98 (0.73–1.50)	50	0.79 (0.61–1.22)	46	1.28 (0.89–1.83)	**0.017**	39	0.76 (0.61–1.06)	11	0.89 (0.63–2.82)	0.163	3	1.07 (0.79–1.38)	13	1.79 (1.68–2.25)	**0.008**
Urea (mg/dL)	96	45.1 (33.4–63.8)	50	44.8 (31.6–63.8)	46	45.4 (35.5–62.9)	0.990	39	41.0 (28.5–59.9)	11	56.6 (35.6–103.0)	0.106	33	43.6 (33.3–50.9)	13	62.9 (48.7–68.4)	**0.017**
Potassium (mmol/L)	98	4.1 (3.8–4.3)	52	4.0 (3.8–4.2)	46	4.1 (4.0–4.4)	0.084	41	3.9 (3.7–4.2)	11	4.3 (4.1–4.5)	0.053	33	4.1 (4.0–4.3)	13	4.2 (4.0–4.5)	0.423
Bilirubin (mg/dL)	93	0.61 (0.35–0.94)	49	0.45 (0.32–0.69)	44	0.84 (0.51–1.30)	**0.006**	39	0.45 (0.32–0.76)	10	0.48 (0.37–0.56)	0.785	32	0.77 (0.51–1.28)	12	0.90 (0.61–1.42)	0.496
AST (U/L)	90	43 (27–90)	49	36 (24–52)	41	75 (42–181)	**<0.001**	39	37 (24–54)	10	28 (19–48)	0.323	30	78 (42–182)	11	70 (29–111)	0.955
ALT (U/L)	92	37 (20–85)	49	26 (19–45)	43	55 (30–199)	**0.001**	39	35 (17–47)	10	21 (19–26)	0.309	31	61 (32–206)	12	38 (28–158)	0.953
Lipase (U/L)	90	86 (61–207)	47	80 (50–146)	43	104 (64–357)	**0.031**	38	83 (50–146)	9	66 (49–82)	0.351	31	94 (63–297)	12	201 (72–541)	0.357
CRP (mg/dL)	96	150 (90–218)	50	140 (87–215)	46	165 (90–223)	0.577	39	148 (85–215)	11	114 (87–218)	0.761	33	175 (92–223)	13	155 (81–214)	0.678
PCT (µg/L)	69	0.63 (0.20–2.29)	36	0.30 (0.15–0.87)	33	1.24 (0.40–3.00)	**0.003**	28	0.26 (0.12–0.62)	8	0.52 (0.27–1.20)	0.304	24	1.46 (0.52–2.81)	9	0.99 (0.40–7.04)	0.952
**Hematology**																	
Hemoglobin (g/dL)	98	8.85 (8.10–10.1)	52	8.90 (8.20–10.6)	46	8.80 (7.80–9.60)	**0.048**	41	8.90 (8.10–10.8)	11	8.90 (8.30–9.70)	0.631	33	8.80 (7.80–9.60)	13	8.80 (8.50–9.50)	0.543
WBC (10^9^/L)	96	11.5 (8.3–14.3)	50	12.5 (10.3–15.4)	46	9.4 (6.3–13.0)	**0.005**	39	8.9 (8.1–10.8)	11	12.6 (7.5–13.8)	0.573	33	9.9 (7.9–12.8)	13	7.3 (4.8–14.9)	0.510
Thrombocytes (10^9^/L)	96	162 (106–250)	50	203 (142–275)	46	123 (82–195)	**<0.001**	39	198 (142–279)	11	229 (136–252)	0.963	33	133 (84–195)	13	103 (81–138)	0.386
INR	96	1.07 (1.00–1.12)	50	1.04 (0.99–1.10)	46	1.10 (1.04–1.19)	**0.003**	39	1.04 (1.00–1.10)	11	1.01(0.95–1.12)	0.606	33	1.07 (1.02–1.16)	13	1.12 (1.07–1.19)	0.271
**Vital signs**																	
Temperature (°C)	96	37.1 (36.8–37.7)	50	37.0 (36.7–37.5)	46	37.2 (36.8–37.8)	0.157	39	37.0 (36.6–37.6)	11	37.1 (36.7–37.5)	0.892	33	37.3 (36.9–37.8)	13	37.1 (36.8–38.0)	0.817
Respiratory rate (1/min)	98	19 (16–22)	52	18 (16–22)	46	20 (16–23)	0.390	41	18 (16–21)	11	20 (16–27)	0.224	33	20 (18–22)	13	19 (16–24)	0.985
Horovitz index (mmHg)	98	280 (220–343)	52	295 (224–353)	46	271 (214–326)	0.276	41	302 (234–357)	11	215 (183–295)	0.271	33	286 (227–326)	13	220 (188–300)	0.294
Shock index	98	0.70 (0.61–0.88)	52	0.66 (0.54–0.82)	46	0.80 (0.65–0.93)	**<0.001**	41	0.66 (0.55–0.82)	11	0.62 (0.49–0.83)	0.856	33	0.80 (0.66–0.89)	13	0.76 (0.64–1.01)	0.495
**Clinical scores**																	
RASS	97	−3 (−5–0)	51	−1 (−3–0)	46	−5 (−5–−3)	**<0.001**	41	−1 (−3–0)	10	0 (−1–1)	0.084	33	−5 (−5–−3)	13	−4 (−5–−1)	0.423
TISS	98	18 (10–22)	52	12 (10–18)	46	22 (18–23)	**<0.001**	41	10 (10–18)	11	13 (10–18)	0.386	33	22 (18–23)	13	23 (22–27)	**0.028**
SAPS II	98	35 (28–43)	52	35 (29–42)	46	35 (26–45)	0.975	41	34 (28–41)	11	39 (35–45)	0.110	33	30 (23–38)	13	45 (40–51)	**<0.001**
SOFA	99	8 (5–11)	53	6 (4–7)	46	12 (10–13)	**<0.001**	42	5 (4–7)	11	6 (5–8)	**0.040**	33	11 (10–12)	13	13 (12–15)	**0.003**
**Hypoxia biomarker**																	
Lactate (mmol/L)	98	1.0 (0.7–1.6)	52	0.9 (0.6–1.1)	46	1.3 (0.8–1.9)	**0.004**	41	0.8 (0.6–1.2)	11	1.1 (0.6–1.1)	0.543	33	1 (0.8–1.5)	13	1.6 (1.4–3.0)	**0.031**

Results are given as median (interquartile range, IQR) or number (%); significant results are highlighted in bold. Abbreviations: BOD = burden of organ dysfunction; *N* = number of patients included; *n* = number of determinations for the variable; insuff. = insufficiency; ARDS = acute respiratory distress syndrome; ICU = intensive care unit; LOS = length of stay; AST = aspartate aminotransferase; ALT = alanine aminotransferase; CRP = C-reactive protein; PCT = procalcitonin; WBC = white blood cell; INR = international normalized ratio; RASS = Richmond Agitation and Sedation Score; TISS = Therapeutic Intervention Severity Score; SAPS = Simplified Acute Physiology Score; SOFA = Sequential Organ Failure Assessment Score.

**Table 2:** Initial values and kinetic parameters of serially determined progranulin plasma concentrations (ng/mL) grouped by burden of organ dysfunction (BOD) and mortality.

Total Cohort (SOFA 3–18)				
	Total Group (*N* = 99)	Survivors (S) (*N* = 75)	Non-Survivors (NS) (*N* = 24)	S vs. NS
Initial/n	45.6 (36.4 62.5)/99	44.1 (36.4 57.6)/75	47.7 (36.5 71.1)/24	0.516
Maximum/n	52.5 (41.8 68.8)/99	48.7 (41.3 65.5)/75	65.5 (50.5 82.9)/24	**0.005**
Mean/n	42.9 (35.3 55.0)/1257	40.8 (34.5 54.0)/955	53.3 (41.3 62.7)/302	**0.010**
NAS/n	43.0 (34.6 55.5)/1257	40.7 (33.7 53.0)/955	52.2 (38.5 62.6)/302	**0.011**
	**Low BOD (SOFA ≤ 8)**			
	**(*N* = 53)**	**(*N* = 42)**	**(*N* = 11)**	
Initial/n	39.2 (33.3 48.3)/53	38.9 (33.3 47.0)/42	39.2 (36.0 51.7)/11	0.539
Maximum/n	44.5 (37.8 59.6)/53	43.0 (36.6 52.5)/42	62.1 (48.2 72.7)/11	**0.001**
Mean/n	38.5 (33.0 46.8)/610	36.2 (30.9 43.3)/467	48.8 (36.8 58.5)/143	**0.010**
NAS/n	37.3 (31.4 45.7)/610	36.1 (29.7 43.4)/467	48.8 (37.0 58.4)/143	**0.016**
	**High BOD (SOFA > 8)**			
	**(*N* = 46)**	**(*N* = 33)**	**(*N* = 13)**	
Initial/n	54.6 (44.6 72.3)/46	54.4 (44.1 72.0)/33	67.3 (41.8 75.7)/13	0.836
Maximum/n	65.5 (49.2 80.8)/46	63.6 (48.7 79.3)/33	67.3 (54.3 85.8)/13	0.435
Mean/n	53.1 (38.5 63.0)/647	46.7 (38.3 62.4)/488	57.3 (47.5 66.8)/159	0.289
NAS/n	50.9 (38.8 62.0)/647	46.0 (38.1 61.7)/488	55.5 (46.6 66.7)/159	0.257

In the total cohort, the range of the initial SOFA scores was 3 to 18. Patients with an initial SOFA ≤ 8 and SOFA > 8 represented patients with low and high burden of organ dysfunction (BOD), respectively. The initial value refers to the first time point of determination, i.e., at the patients’ study inclusion. The maximum and mean give the highest or calculated averaged value of all collected values within a total of up to 14 eight-hour periods, within the total study period of up to 112 h. The normalized area scores (NAS) were obtained by plotting the values of progranulin for each patient within the respective period, followed by the division of the areas under the curves by the period of observation. The results are given as median (interquartile range, IQR); significant results by the Mann–Whitney U test between survivors and non-survivors are highlighted in bold. Abbreviations: N = number of patients included; n = number of determinations for each parameter; NAS: normalized area score.

**Table 3:** Univariate logistic regression analyses of initial values and kinetic parameters of serially determined progranulin plasma concentrations for prediction of in-hospital mortality grouped by burden of organ dysfunction (BOD).

Total Cohort (SOFA 3–18, *N* = 99)					
	Coefficient Means (SE)	Odds Ratio Means (95% CI)	*p*-Value	AUROC Means (SE)	*p*-Value
Initial	0.010 (0.012)	1.010 (0.985–1.035)	0.443	0.544 (0.072)	0.516
Maximum	0.027 (0.012)	1.028 (1.004–1.052)	**0.020**	0.691 (0.063) *	**0.005**
Mean	0.038 (0.016)	1.038 (1.006–1.072)	**0.021**	0.676 (0.064) *	**0.010**
NAS	0.039 (0.016)	1.040 (1.007–1.074)	**0.017**	0.673 (0.065) *	**0.011**
	**Low BOD (SOFA ≤ 8, *N* = 53)**				
Initial	0.023 (0.022)	1.024 (0.980–1.069)	0.292	0.561 (0.097)	0.539
Maximum	0.082 (0.028)	1.086 (1.027–1.148)	**0.004**	0.815 (0.068) *	**0.001**
Mean	0.097 (0.037)	1.102 (1.025–1.184)	**0.008**	0.753 (0.075) *	**0.010**
NAS	0.089 (0.035)	1.093 (1.021–1.170)	**0.011**	0.738 (0.080) *	**0.016**
	**High BOD (SOFA > 8, *N* = 46)**				
Initial	−0.004 (0.017)	0.996 (0.963–1.030)	0.817	0.520 (0.102)	0.836
Maximum	0.002 (0.016)	1.002 (0.971–1.034)	0.899	0.575 (0.099)	0.435
Mean	0.015 (0.021)	1.015 (0.975–1.057)	0.473	0.601 (0.096)	0.289
NAS	0.018 (0.021)	1.019 (0.978–1.061)	0.380	0.608 (0.097)	0.257
	**∆ AUROC = AUROC_LowBOD_ − AUROC_HighBOD_** **Means (SE)**				***p*-value**
Initial	0.041 (0.141)				0.772
Maximum	0.240 (0.121)				**0.047**
Mean	0.152 (0.122)				0.214
NAS	0.130 (0.126)				0.302

In the total cohort, the range of the initial SOFA scores was 3 to 18. Patients with an initial SOFA ≤ 8 and SOFA > 8 represent patients with low and high burden of organ dysfunction (BOD), respectively. Regression coefficient, odds ratios, and AUROC values are given as mean and standard error or the confidence interval is shown. *p*-values indicating significant results of univariate logistic regression analyses are given in bold. *p*-values of AUROC analyses reaching the level of statistical significance are also given in bold. The AUROC values are highlighted in bold only, if both the respective logistic regression and the AUROC analysis reached the level of statistical significance and the AUROC value was considered at least acceptable (AUROC ≥ 0.7 and <0.8) or excellent (AUROC ≥ 0.8 and <0.9) according to the classification of AUROC values by Hosmer and Lemeshow [26]. The AUROC values of kinetic progranulin parameters showed statistically significant discrimination for mortality in the total cohort, with even higher values in the subgroup of patients with low BOD. In both groups, the AUROC values derived from all kinetic progranulin parameters were significantly greater than those based on the initial values as indicated (* *p* ≤ 0.01). By contrast, none of the progranulin parameters showed significant discriminatory power for mortality in the subgroup of patients with high BOD. Intergroup differences in the AUROC values were calculated as Δ AUROC = AUROC_Low BOD_ − AUROC_High BOD_. The calculated intergroup differences in the AUROC values reached the level of significance for progranulin maximum values. Abbreviations: AUROC = area under the receiver operator characteristic curve. NAS = normalized area score. For further explanation of initial and kinetic parameters (Max, Mean, NAS) see Legend of Table 2.

**Table 4:** Sensitivity analysis: Univariate logistic regression analyses of initial values and kinetic parameters of serially determined progranulin plasma concentrations for prediction of in-hospital mortality grouped by SOFA cutoff value of 6 or 10.

	Coefficient Means (SE)	Odds Ratio Means (95% CI)	*p*-Value	AUROC Means (SE)	*p*-Value
	**SOFA ≤ 6, *N* = 34**				
Initial	0.033 (0.026)	1.033 (0.983–1.086)	0.203	0.613 (0.129)	0.391
Maximum	0.078 (0.032)	1.081 (1.016–1.150)	**0.014**	0.854 (0.080) *	**0.007**
Mean	0.124 (0.049)	1.132 (1.027–1.27)	**0.012**	0.827 (0.085) *	**0.013**
NAS	0.117 (0.047)	1.124 (1.025–1.233)	**0.013**	0.827 (0.085) *	**0.013**
	**SOFA > 6, *N* = 65**				
Initial	−0.003 (0.015)	0.997 (0.968–1.027)	0.857	0.501 (0.087)	0.988
Maximum	0.010 (0.014)	1.010 (0.983–1.038)	0.461	0.603 (0.081) *	0.202
Mean	0.016 (0.019)	1.017 (0.980–1.054)	0.374	0.592 (0.082)	0.256
NAS	0.019 (0.019)	1.019 (0.982–1.057)	0.320	0.595 (0.082) *	0.433
	**SOFA ≤ 10, *N* = 69**				
Initial	−0.002 (0.022)	0.998 (0.957–1.041)	0.931	0.454 (0.096)	0.618
Maximum	0.043 (0.020)	1.044 (1.004–1.085)	**0.029**	0.691(0.092) *	**0.039**
Mean	0.044 (0.027)	1.045 (0.990–1.102)	0.109	0.656 (0.090) *	0.090
NAS	0.040 (0.027)	1.041 (0.988–1.097)	0.132	0.642 (0.093) *	0.125
	**SOFA > 10, *N* = 30**				
Initial	−0.008 (0.019)	0.992 (0.955–1.031)	0.671	0.495(0.110)	0.966
Maximum	−0.005 (0.019)	0.995 (0.960–1.033)	0.807	0.521(0.109)	0.849
Mean	0.009 (0.025)	1.009 (0.960–1.060)	0.733	0.565 (0.108)	0.553
NAS	0.015 (0.026)	1.015 (0.966–1.068)	0.553	0.574 (0.108)	0.498
**∆ AUROC = AUROC_SOFA≤6_ − AUROC_SOFA>6_** Means (SE)					
Initial	0.100 (0.157)				0.525
Maximum	0.233 (0.115)				**0.042**
Mean	0.211 (0.118)				0.074
NAS	0.208 (0.119)				0.080
**∆ AUROC = AUROC_SOFA≤10_ − AUROC_SOFA>10_** Means (SE)					
Initial	−0.048 (0.150)				0.749
Maximum	0.183 (0.147)				0.212
Mean	0.115 (0.143)				0.421
NAS	0.089 (0.145)				0.615

Regression coefficient, odds ratios, and AUROC values are given as mean and standard error or the confidence interval is shown. *p*-values indicating significant results of univariate logistic regression analyses are given in bold. *p*-values of AUROC analyses reaching the level of statistical significance are also given in bold. The AUROC values are highlighted in bold only, if both the respective logistic regression and the AUROC analysis reached the level of statistical significance and the AUROC value was considered to be at least acceptable (AUROC ≥ 0.7 and <0.8) or excellent (AUROC ≥ 0.8 and <0.9) according to the classification of AUROC values by Hosmer and Lemeshow [26]. The AUROC values of kinetic progranulin parameters showed statistically significant discrimination for mortality in the subgroup of patients with SOFA ≤ 6. In both groups below the respective thresholds (≤6 and ≤10), the AUROC values derived from all kinetic progranulin parameters were significantly greater than those based on the initial values as indicated (* *p* ≤ 0.01). By contrast, none of the progranulin parameters showed significant discriminatory power for mortality in the subgroup of patients with SOFA > 6 and >10, respectively. Intergroup differences in the AUROC values were calculated as Δ AUROC = AUROC_SOFA≤6_ − AUROC_SOFA>6_ and AUROC_SOFA≤10_ − AUROC_SOFA>10_, respectively. The calculated intergroup differences in the AUROC values reached the level of significance for progranulin maximum values using the SOFA cutoff of 6. Abbreviations: AUROC = area under the receiver operator characteristic curve. NAS = normalized area score. For further explanation of initial and kinetic parameters (Max, Mean, NAS) see Legend of Table 2.

## Data Availability

The data presented in this study are available upon request from the corresponding author due to patient privacy.

## Supplementary Materials

The following supporting information can be downloaded at https://www.mdpi.com/article/10.3390/biomedicines14040744/s1, Table S1, Univariate logistic regression analyses of initial values and kinetic parameters of serially determined SOFA scores for prediction of in-hospital mortality grouped by burden of organ dysfunction (BOD); Table S2, Univariate logistic regression analyses of initial values and kinetic parameters of serially determined C reactive protein (CRP) values for prediction of in-hospital mortality grouped by burden of organ dysfunction (BOD); Table S3, Correlation matrix for maximum values of progranulin plasma concentrations and parameters of organ dysfunction, inflammation, coagulation, and hypoxia in patients grouped by burden of organ dysfunction (BOD); Figure S1, Distribution of maximum progranulin values in survivors and non-survivors within the low BOD group.
