# Supplementary material for: Progranulin Is a Useful Biomarker to Predict Mortality in ICU Patients with Low Burden of Organ Dysfunction
Source: Biomedicines. 2026 Mar 24;14(4):744. doi: 10.3390/biomedicines14040744 (PMC13113082; doi:10.3390/biomedicines14040744)
Supplement: Supplementary file 1 [file biomedicines-14-00744-s001.zip › Figure S1.pdf]

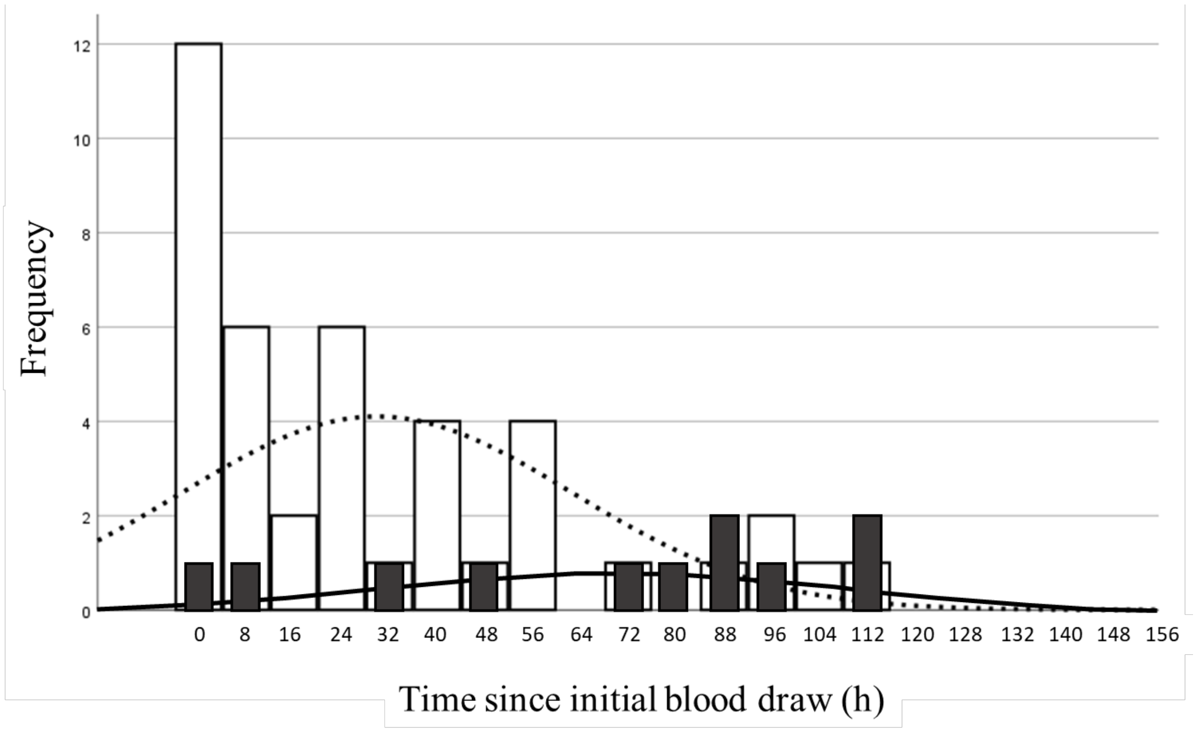

Supplementary Figure S1. Distribution of maximum progranulin values across the study period (0-112h) in survivors and non survivors within the low BOD group. Histograms of the time points at which survivors (white bars) and non survivors (black bars) within the low BOD group (SOFA ≤ 8) reached their maximum progranulin values. In survivors, the distribution of maximum value time points was positively (right)skewed (mean (SE): 1.1 (0.365)), whereas in non survivors it was negatively (left)skewed (mean (SE): –0.648 (0.661)). The dashed and solid lines represent the corresponding normal distributions for survivors and non survivors. Although the two distributions differed significantly (Mann–Whitney U test, p = 0.009), substantial overlap remained. For further details, see Section 3.6 in the results section of the main manuscript.
