# Supplementary material for: Progranulin Is a Useful Biomarker to Predict Mortality in ICU Patients with Low Burden of Organ Dysfunction
Source: Biomedicines. 2026 Mar 24;14(4):744. doi: 10.3390/biomedicines14040744 (PMC13113082; doi:10.3390/biomedicines14040744)
Supplement: Supplementary file 1 [file biomedicines-14-00744-s001.zip › Table S1.pdf]

Table S1. Univariate logistic regression analyses of initial values and kinetic parameters of serially determined SOFA scores for prediction of in-hospital mortality grouped by burden of organ dysfunction (BOD) (modified from [19]).

Total cohort (SOFA 3 - 18, *N* = 99)					
	**Coefficient** Means (SE)	**Odds Ratio** Means (95% CI)	**p-** value	**AUROC** Means (SE)	**p-value**
Initial	0.184 (0.070))	1.202 (1.049-1.378)	**0.008**	0.669 (0.064)	**0.013**
Maximum	0.227 (0.073)	1.255 (1.088-1.447)	**0.002**	0.695 (0.065)	**0.004**
Mean	0.250 (0.074)	1.285 (1.112-1.484)	**0.001**	**0.727 (0.058)**	**0.001**
NAS	0.289 (0.080)	1.335 (1.141-1.562)	**0.001**	**0.728 (0.059)**	**0.001**
**Low BOD (SOFA ≤ 8, *N* = 53)**					
Initial	0.476 (0.239)	1.609 (1.007–2.571)	**0.047**	**0.701 (0.079)**	**0.041**
Maximum	0.276 (0.144)	1.318 (0.994–1.749)	0.055	0.685 (0.076)	0.061
Mean	0.178 (0.126)	1.195 (0.934–1.529)	0.157	0.741 (0.070)	**0.014**
NAS	0.327 (0.169)	1.386 (0.996–1.929)	0.053	0.723 (0.073)	**0.024**
**High BOD (SOFA > 8, N = 46)**					
Initial	0.537 (0.196)	1.711 (1.165–2.515)	**0.006**	**0.780 (0.071)**	**0.003**
Maximum	0.603 (0.197)	1.828 (1.241–2.691)	**0.002**	**0.815 (0.072)**	**0.001**
Mean	0.541 (0.172)	1.718 (1.228–2.405)	**0.002**	**0.838 (0.066)**	**0.001**
NAS	0.540 (0.172)	1.717 (1.226–2.403)	**0.002**	**0.840 (0.065)**	**0.001**

	**Δ AUROC = AUROC$_{LowBOD}$ - AUROC$_{HighBOD}$** Means (SE)	**p-value**
Initial	0.078 (0.106)	0.460
Maximum	0.130 (0.105)	0.217
Mean	0.097 (0.096)	0.313
NAS	0.117 (0.098)	0.229

In the total cohort, range of initial SOFA scores was 3 to 18. Patients with initial SOFA ≤ 8 and SOFA > 8 represent patients with low and high burden of organ dysfunction (BOD), respectively. Regression coefficient, odds ratio and AUROCs are given as means and standard error or the confidence interval is shown. P-values indicating significant results of univariate logistic regression analyses are given in bold. P-values of AUROC analyses reaching the level of statistical significance are also given in bold. Values of AUROCs are highlighted in bold only, if both the respective logistic regression and AUROC analysis reached the level of statistical significance and the AUROC value was considered at least acceptable (AUROC > 0,7 and < 0,8) or excellent (AUROC ≥ 0,8 and < 0,9) according to the classification of AUROC values by Hosmer and Lemeshow [26]. Intergroup differences in AUROCs were calculated as Δ AUROC = AUROC Low BOD − AUROCHigh BOD. None of the calculated intergroup differences in AUROCs reached the level of significance. Abbreviations: AUROC = area under the receiver operator characteristic curve. NAS = normalized area score. For further explanation of initial and kinetic parameters (Max, Mean, NAS) see Legend of Table 1.
