# Supplementary material for: Progranulin Is a Useful Biomarker to Predict Mortality in ICU Patients with Low Burden of Organ Dysfunction
Source: Biomedicines. 2026 Mar 24;14(4):744. doi: 10.3390/biomedicines14040744 (PMC13113082; doi:10.3390/biomedicines14040744)
Supplement: Supplementary file 1 [file biomedicines-14-00744-s001.zip › Table S2.pdf]

Table S2. Univariate logistic regression analyses of initial values and kinetic parameters of serially determined C reactive protein (CRP) values for prediction of in-hospital mortality grouped by burden of organ dysfunction (BOD).

	**Coefficient** Means (SE)	**Odds Ratio** Means (95% CI)	**p-** value	**AUROC** Means (SE)	**p- value**
**Total cohort (SOFA 3 - 18, N = 99)**					
Initial	-0.002 (0.003))	0.998 (0.993-1.003)	0.504	0.466 (0.067)	0.623
Maximum	0.003 (0.002)	1.003 (0.999-1.008)	0.138	0.587 (0.065)*	0.200
Mean	0.002 (0.003)	1.002 (0.997-1.007)	0.423	0.562 (0.065)	0.363
NAS	0.003 (0.003)	1.003 (0.998-1.008)	0.244	0.565 (0.067)	0.434
**Low BOD (SOFA ≤ 8, N = 53)**					
Initial	-0.002 (0.004)	0.998 (0.991–1.005)	0.619	0.468 (0.093)	0.745
Maximum	0.000 (0.003)	1.000 (0.994–1.007)	0.904	0.534 (0.093)	0.728
Mean	-0.003 (0.004)	0.997 (0.998–1.005)	0.439	0.486 (0.086)	0.884
NAS	-0.004 (0.005)	0.996 (0.988–1.005)	0.437	0.481 (0.084)	0.849
**High BOD (SOFA > 8, N = 46)**					
Initial	-0.002 (0.004)	0.998 (0.991–1.006)	0.645	0.459 (0.099)	0.669
Maximum	0.006 (0.003)	1.006 (0.999–1.012)	0.094	0.631 (0.092)	0.172
Mean	0.008 (0.004)	1.008 (0.999–1-017)	0.073	0.648 (0.093)*	0.121
NAS	0.009 (0.004)	1.009 (1.000–1.017)	**0.043**	0.662 (0.099)*	0.091

	**Δ AUROC = AUROC$_{LowBOD}$ - AUROC$_{HighBOD}$** Means (SE)	**p- value**
Initial	0.009 (0.135)	0.949
Maximum	-0.096 (0.130)	0.461
Mean	-0.162 (0.127)	0.200
NAS	-0.181 (0.130)	0.163

In the total cohort, range of initial SOFA scores was 3 to 18. Patients with initial SOFA ≤ 8 and SOFA > 8 represent patients with low and high burden of organ dysfunction (BOD), respectively. Regression coefficient, odds ratio and AUROCs are given as mean and standard error or the confidence interval is shown. P-values indicating significant results of univariate logistic regression analyses are given in bold. P-values of AUROC analyses reaching the level of statistical significance are also given in bold. Values of AUROCs are highlighted in bold only, if both the respective logistic regression and AUROC analysis reached the level of statistical significance and the AUROC value was considered at least acceptable (AUROC > 0,7 and < 0,8) or excellent (AUROC ≥ 0,8 and < 0,9) according to the classification of AUROC values by Hosmer and Lemeshow [26]. None of the AUROCs of the kinetic CRP parameters demonstrated statistically significant discrimination for mortality in any of the groups analyzed. AUROCs derived from maximum CRP in the total cohort, and from mean and NAS CRP in the subgroup of patients with high BOD were significantly greater than those based on the initial values as indicated (* $p \leq 0.01$). Intergroup differences in AUROCs were calculated as Δ AUROC = AUROC$_{Low\ BOD}$ − AUROC$_{High\ BOD}$. None of the calculated intergroup differences in AUROCs reached the level of significance. Abbreviations: AUROC = area under the receiver operator characteristic curve. NAS = normalized area score. For further explanation of initial and kinetic parameters (Max, Mean, NAS) see Legend of Table 2.
