# Supplementary material for: Progranulin Is a Useful Biomarker to Predict Mortality in ICU Patients with Low Burden of Organ Dysfunction
Source: Biomedicines. 2026 Mar 24;14(4):744. doi: 10.3390/biomedicines14040744 (PMC13113082; doi:10.3390/biomedicines14040744)
Supplement: Supplementary file 1 [file biomedicines-14-00744-s001.zip › Table S3.pdf]

Table S3. Correlation matrix for maximum values of plasma concentrations of progranulin and parameters of organ dysfunction, inflammation, coagulation and hypoxia in patients grouped by burden of organ dysfunction

Maximum	SOFA	CRP	Temp	INR	Platelets	SAH	Lactate
Progranulin (*r*/p/*n*)	**All (SOFA 3 - 18, *N* = 99)**						
	**0.563**<br><0.001/99	**0.307**<br>0.002/98	**0.250**<br>0.014/95	**0.473**<br><0.001/94	**-0.260**<br>0.011/96	**0.399**<br><0.001/99	**0.408**<br><0.001/98
	**Low BOD (SOFA ≤ 8, *N* = 53)**						
	**0.402**<br>0.003/53	**0.287**<br>0.039/52	0.007<br>0.961/50	**0.489**<br><0.001/51	**-0.364**<br>0.009/50	**0.403**<br>0.003/53	**0.375**<br>0.006/52
SOFA (*r*/p/*n*)	**All (SOFA 3-18, *N* = 99)**						
	1.0	0.051<br>0.616/98	0.121<br>0.242/95	**0.307**<br>0.002/96	**-0.402**<br>0.001/96	**0.308**<br>0.002/99	**0.424**<br>0.001/98
	**Low BOD (SOFA ≤ 8, *N* = 53)**						
	1.0	0.229<br>0.102/52	0.227<br>0.113/50	0.233<br>0.099/51	-0.105<br>0.469/50	0.159<br>0.254/53	0.203<br>0.148/52

Correlations were calculated by Pearson analyses. Values of correlation coefficients are highlighted in bold, if p values reached the level of significance. Abbreviations: N gives the number of patients per group, n is the number of data pairs of maximum values of each parameter. SOFA = Sequential Organ Failure Assessment, CRP = C-reactive protein, Temp = temperature, INR, international normalized ratio, SAH = S-adenosylhomocysteine.
